# Iridium-Catalyzed Hydrocarboxylation of Olefins with CO_2_ and H_2_

**DOI:** 10.3390/molecules30071599

**Published:** 2025-04-03

**Authors:** Yang Li, Ying Wang, Longbo Zhang, Yanru Zhang, Jia Guo, Yanyan Wang, Chenglong Yu, Jun He, Zhenpeng Wang, Juanjuan Han, Qian Li, Tianbin Wu, Qingli Qian, Buxing Han

**Affiliations:** 1 Beijing National Laboratory for Molecular Sciences, Key Laboratory of Colloid, Interface and Thermodynamics, Center for Carbon Neutral Chemistry, Institute of Chemistry, Chinese Academy of Sciences, Beijing 100190, China; liyangly1102@163.com (Y.L.); wangying2016@iccas.ac.cn (Y.W.); zlb_iccas@163.com (L.Z.); zhangyanru.ripp@sinopec.com (Y.Z.); gjxzjz@iccas.ac.cn (J.G.); 17860759737@163.com (Y.W.); yuyucl@iccas.ac.cn (C.Y.); hejun_sunflower@163.com (J.H.); wtb@iccas.ac.cn (T.W.); 2 School of Chemical Sciences, University of Chinese Academy of Sciences, Beijing 100049, China; 3 Center for Physicochemical Analysis Measurements, Institute of Chemistry, Chinese Academy of Sciences, Beijing 100190, China; wang_82713@iccas.ac.cn (Z.W.); hjuan@iccas.ac.cn (J.H.); qianlee@iccas.ac.cn (Q.L.); 4 Shanghai Key Laboratory of Green Chemistry and Chemical Processes, School of Chemistry and Molecular Engineering, East China Normal University, Shanghai 200062, China

**Keywords:** carbon dioxide, carboxylic acids, hydrogen, green chemistry, homogeneous catalysis

## Abstract

CO_2_ is a greenhouse gas and a nontoxic, easily available and renewable C_1_ feedstock. H_2_ is a clean and cheap reductant that can be obtained from renewable energy. Olefins are platform chemicals that can be produced from a variety of raw materials such as petroleum, coal and renewable biomass. The production of carboxylic acids by combining olefins, CO_2_ and H_2_ is a sustainable and very promising protocol. However, only a few advances in this topic have been achieved because novel catalysts need to be developed. In this work, we demonstrate that a simple iridium-based catalyst could efficiently promote the synthesis of C_2+_ carboxylic acids via the reaction of olefins with CO_2_ and H_2_. The reaction was effectively accelerated by a simple iridium-based catalytic system at 170 °C, which may be applied to various olefin substrates. The catalytic mechanism was studied through a series of control experiments. The findings contribute to advancing the sustainable production of valuable products by the reaction of renewable CO_2_ and green H_2_ with platform chemicals.

## 1. Introduction

Carbon dioxide is a major greenhouse gas and a renewable C_1_ resource. The conversion of CO_2_ into value-added products is an important aspect of carbon neutrality. Currently, significant developments have been accomplished in the synthesis of various chemicals utilizing CO_2_ as the feedstock [1,2,3,4,5,6,7,8,9,10,11,12,13]. CO_2_ is the final product of the combustion of organics, where the C is in the highest oxidation state, and reduction is usually required in case of CO_2_ utilization. As for the carboxylic acid products, at the present stage, formic acid can be readily produced from CO_2_ and H_2_, which has been extensively investigated, and great advances have been made [14,15,16,17,18]. Currently, the fabrication of C_2+_ carboxylic acids using CO_2_ usually requires expensive or air/water-sensitive substrates and/or reductants. [19,20,21,22,23,24,25,26,27,28,29,30]. For example, the substrates with C-B, C-Br or even C-Zn bonds were extensively studied to react with CO_2_, where C_1_-elongating carboxylic acids could be produced. Furthermore, many examples of C_2+_ carboxylic acids syntheses were realized by coupling reactions between CO_2_ and unsaturated organics such as alkynes, dienes, allenes or olefins with Pd, Ni or Fe catalysts, where metal-based reducing agents such as Mn/Zn powder, ZnR_2_, AlR_3_ and silanes were required.

H_2_ is a clean and cheap reductant, which can be used to reduce CO_2_ when the target products are CO, hydrocarbons, alcohols, etc. [3,4,5,6,7]. However, only sporadic reports on the production of higher carboxylic acids using CO_2_ and H_2_ can be found. In a pioneering work, higher carboxylic acids were successfully synthesized by a reaction of olefins with CO_2_ and H_2_ using a four-component catalytic system containing a [RhCl(CO)_2_]_2_ catalyst, PPh_3_ ligand, CH_3_I promoter and p-TsOH·H_2_O as an acidic additive [31]. Later, some other routes to fabricate C_2+_ carboxylic acids were developed, where CO_2_ and H_2_ reacted with various oxygenates, including alcohols, polyols, ethers, ketones, aldehydes, epoxides and saccharides [32,33,34,35,36,37,38,39]. Olefins are a type of easily available and widely used platform chemicals that can be obtained from various sources such as petroleum, coal and renewable biomass. H_2_ can be manufactured from the electrolysis of water with renewable electricity. Without a doubt, the synthesis of C_2+_ carboxylic acids from olefins, CO_2_ and H_2_ is a very promising route, and more research on this topic is still highly desirable. However, new catalysts in these reactions have seldom been reported so far. Iridium catalysts are common alternatives to drive the formation of carboxylic acids by carbonylation [34,35,36,40]. Herein, we report a new and simpler Ir catalytic system to accelerate this kind of reaction.

## 2. Results and Discussion

### 2.1. The Catalytic System

To screen the catalytic systems, we selected the hydrocarboxylation of cyclohexene with CO_2_ and H_2_ as a model reaction (Table 1). The cyclohexanecarboxylic acid (ChA) generated in the reaction is much more expensive than the cyclohexene feedstock (Table S1).

The reaction can be effectively accelerated by the catalytic system consisting of an Ir(acac)(CO)_2_ catalyst and LiI promoter in the solvent of acetic acid at 170 °C (Table 1, entry 1). In this condition, cyclohexene was completely consumed, and the yields of ChA could reach 62.8% (Figure S1). The rest of the cyclohexene was turned into cyclohexane (Figure S2). The gaseous byproducts were CO and CH_4_ generated from CO_2_ and H_2_ (Figure S3). The iridium catalyst is necessary, and no carboxylic acid was detected without it. Some other iridium compounds could also catalyze the reaction, but with lower reaction yields, such as Ir(OAc)_3_, IrI_4_, Ir_4_(CO)_12_, Ir(acac)_3_ and IrCl_3_ (Table 1, entries 2–6). The target reaction did not occur when the IrO_2_·2H_2_O or IrCl(CO)(PPh_3_)_2_) were used (Table 1, entries 7 and 8). We also tested other transition metal (Fe, Co, Ni, Rh, Pd) iodides, and they could not accelerate the reaction either (Table S2). Thus, Ir(acac)(CO)_2_ was the suitable catalyst of the reaction.

The promoter is also a necessary catalytic component of the reaction. The reaction did not take place when iodine (I_2_) was used instead of LiI, which confirmed that I^−^ plays an important role in this reaction (Table 1, entry 9). To understand the effect of I^−^, we used LiCl and LiBr as promoters, respectively, but they did not work at all (Table 1, entries 10 and 11). This may be ascribed to the stronger nucleophilicity of I^−^ that helps to promote the C-C bond formation; moreover, as a soft base, I^−^ may form a more stable active center with the Ir cation than other halide anions [41]. To seek a possible better cation of the promoter, we substituted the Li^+^ of the promoter with Na^+^ and K^+,^ respectively (Table 1, entries 12 and 13). NaI as a promoter may operate at lower efficiency, while the KI did not operate at all. Thus, smaller Li^+^ cation is more effective in combination with the I^−^. This may be ascribed to the stronger Lewis acidity of Li^+^. The methyl iodide (CH_3_I) was also tested, but the results were unsatisfactory (Table 1, entry 14). Therefore, LiI was the appropriate promoter of the reaction.

The solvent also has an important effect on the reaction. When propionic acid was used as solvent instead of acetic acid, the reaction could also proceed but with a lower reaction yield (Table 1, entry 15). The pKa acidities of acetic acid (4.76) and propionic acid (4.88) are similar, while the acetic acid has better solubility for the catalytic components, especially LiI. Various other organic solvents, inorganic solvents and their mixtures were screened to give a deeper understanding of the solvent effects, and the results are displayed in Table S3. When water and some common organics (H_2_O, DMSO, DMI, NMP) were applied as solvents instead of acetic acid, the aimed reaction could not take place in them. No ChA was detected when aqueous sulfuric acid or a hydrochloric acid solution was used as the reaction solvent. We further tried the acidic mixed solvents such as HCl(aq)/NMP and CF_3_COOH/H_2_O. Before the reaction, we tested the acidities of acetic acid and the mixed solvents using the pH indicator paper. The HCl(aq)/NMP has a similar acidity with acetic acid, while the acidity of CF_3_COOH/H_2_O is much stronger than that of acetic acid. However, only a small amount of ChA was generated when the two mixed solvents were used in the reaction (Table S3, entries 7 and 8). These findings suggest that the reaction yield was simultaneously affected by the organic structure and acidity of the solvent. The organic environment could help to dissolve the olefin substrate and reaction intermediates, while the acidic condition could facilitate the catalytic process.

**Figure 1:**
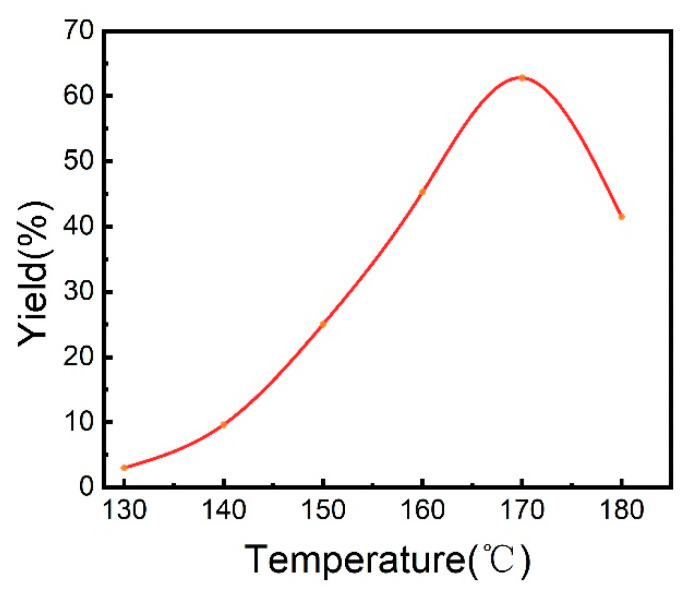
The impact of temperature on the reaction of cyclohexene with CO_2_ and H_2_. Other conditions were the same as those of entry 1 in Table 1.

The impact of reaction temperature was appraised, and the results are shown in Figure 1. The reaction started to occur at 130 °C, and a small amount of ChA was detected. The yield of product increased with rising temperature and reached maximum at 170 °C. Further enhancing the temperature may cause the severe conversion of cyclohexene to cyclohexane before the desired transformation. The dosages of catalyst and promoter may also influence the results of the reaction (Table S4 and S5). The yield of ChA rose with the increasing dosages of the Ir(acac)(CO)_2_ and LiI, but excessive usage of them may cause an opposite effect. The suitable volume of the reaction solvent was 0.6 mL acetic acid, which could engender the optimal environment for the reaction (Table S6). We also investigated the impact of the pressures of CO_2_ and H_2_ (Table S7). Both CO_2_ and H_2_ are necessary for the reaction, and the desired product could not form without anyone of them. The relative pressures of the reactant gases exerted a remarkable role on the yield of the reaction, and 5 MPa CO_2_ and 1 MPa H_2_ were fit for the reaction. As anticipated, the yield of ChA continued to rise with time, but it became unobvious when the time was longer than 14 h (Table S8). In short, the superior and economic reaction result was engendered at the conditions in entry 1 of Table 1.

### 2.2. The Mechanistic Study

To study the intermediates of the reaction, we analyzed the liquid sample after 1h of the reaction, where iodocyclohexane and cyclohexyl acetate derived from cyclohexene were observed (Figure S4). Obvious CO was generated via a Reverse Water Gas Shift Reaction (CO_2_ + H_2_ ⇌ CO + H_2_O, RWGS) when we analyzed the gaseous products at the same time (Figure S5). To unravel the further action of these intermediates during the reaction, some additional control experiments were implemented. When CO of different pressures was used instead of CO_2_ and H_2_ to react with cyclohexene, high yields of ChA could be obtained (Table 2). The lower pressure of CO at 0.5 MPa or 1 MPs was similar to the partial pressure of CO in the hydrocarboxylation reaction, which was in situ generated from CO_2_ and H_2_ by an rWGS reaction. We also carried out the mutual reactions of cyclohexyl acetate and iodocyclohexane with CO_2_/H_2_ or CO, respectively (Table 3). The results further showed that CO, iodocyclohexane and cyclohexyl acetate were all reactive intermediates of the desired reaction.

**Figure 2:**
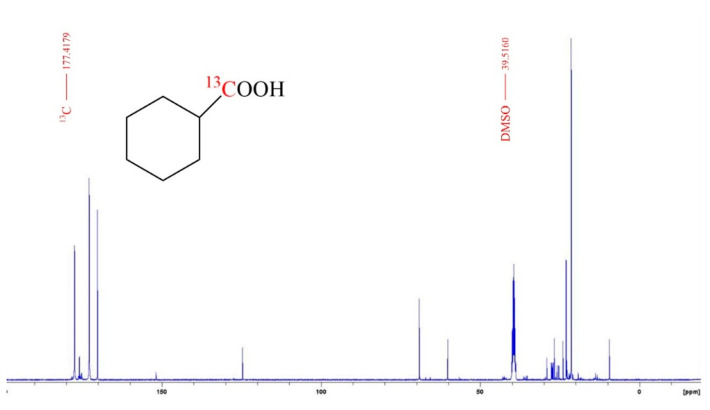
The ^13^C NMR spectra of the liquid sample after the ^13^CO_2_ labeling test. The conditions were the same as those of entry 1 in Table 1 except that 4 MPa ^13^CO_2_ was used instead of CO_2_.

To uncover more about the reaction path, we performed a series of isotope tracer studies. The ^13^CO_2_ labeling test was carried out, and the solution after the reaction was analyzed by ^13^C-NMR and GC-MS. The GC-MS result showed that the C atom of the CO_2_ entered the ChA molecule (Figure S6). The ^13^C-NMR analysis further confirmed that the C atom of the CO_2_ partook in the carboxyl group of the ChA (Figure 2). We added a small amount of H_2_^18^O to carry out the experiment, and the result showed that -OH from the H_2_O solvent was involved in the construction of carboxyl groups, which is in agreement with the characteristics of a carbonylation step (Figure S7). Significant H-D exchange was observed on the carbon chain in the D_2_ labeling tests (Figure S8), which is similar to the Fischer–Tropsch (FT) synthesis.

**Figure 3:**
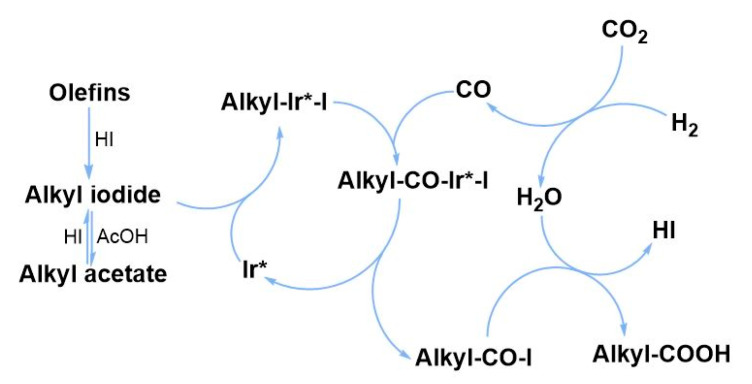
The possible pathway of the reaction of olefin with CO_2_ and H_2_.

**Figure 4:**
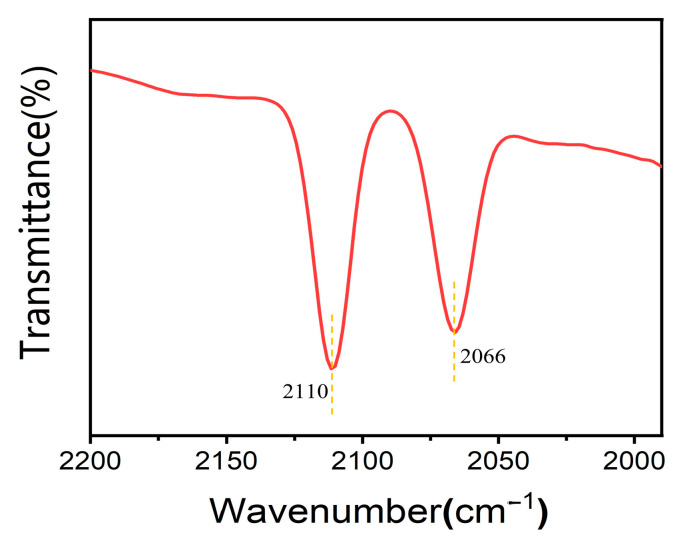
FTIR spectra of the reaction solution. The conditions were the same as those of entry 1 in Table 1.

**Figure 5:**
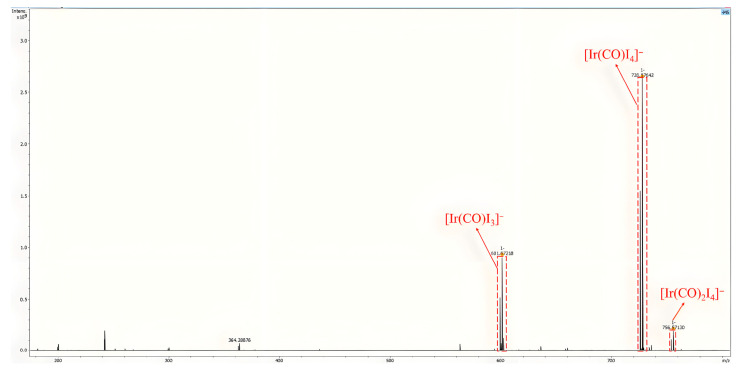
The HR-ESI(-)-MS result of the liquid sample after the reaction of entry 1 in Table 1.

Based on the above results and our former experience [34], we proposed a possible pathway of the reaction, as depicted in Figure 3. Olefins as substrates can form alkyl iodide directly with in situ generated HI. In the acetic acid solvent, mutual transformation of alkyl iodide and alkyl acetate was observed in the control experiment. After the oxidative addition of alkyl iodide to the active Ir center (Ir*), the CO produced through the RWGS reaction is inserted to form the alkyl-CO-Ir*-I. Then, the alkyl-CO-I was formed by reductive elimination from the alkyl-CO-Ir*-I, which was further converted to C_1_-elongated carboxylic acid with the participation of water generated in situ. The FTIR spectra of the solution after the reaction showed two ν(CO) peaks at 2066 cm^−1^ and 2110 cm^−1^ (Figure 4), which demonstrated the formation of cis-[Ir(CO)_2_I_4_]^−^ as the possible major active species [40]. During the HR-ESI(-)-MS test of reaction solution, other notable Ir species, i.e., [Ir(CO)I_x_]^−^ (x = 3–4), were also detected, which should be generated from fragmentation of the cis-[Ir(CO)_2_I_4_]^−^ during the analysis (Figure 5).

### 2.3. The Extension of the Olefin Feedstocks

The iridium-based catalytic system had displayed good performance in the synthesis of ChA with cyclohexene as a substrate. To make certain whether it may apply to other cyclic or linear olefins, we carried out extended reactions (Table 4). The results suggested that moderate yields of different carboxylic acids could be obtained when cyclic, linear or even diene were adopted as the feedstocks. When internal linear olefin was used as the substrate (2-pentene), the terminal carboxylic acid still occupied the highest portion among the acid products (entry 3). This indicated the catalyst possesses strong capability of olefin isomerization during the reaction [39]. The substituents on the olefin substrates may significantly affect the reaction results. We tested a tri-substituted alkene, 2-methyl-2-butene, which is a monomer of 2-pentene. The yield of C_6_ carboxylic acids from 2-methyl-2-butene was 17.0%, which is much lower than that from 2-pentene (49.8%) (entries 3 and 7). In addition, the distributions of the carboxylic acids from 2-methyl-2-butene and 2-pentene were significantly different. We also tried other olefin substrates with different functional groups, such as phenyl or amide groups. When styrene was applied as a substrate, only a little target carboxylic was observed (<1%). Moreover, when some different enecarbamates were utilized, no desired carboxylic acid was observed, and remarkable decomposition of the substrates occurred.

## 3. Materials and Methods

### 3.1. Chemicals and Reagents

Cyclohexene (≥99.5%), heptanoic acid (>98.0%), 2-methylhexanoic acid (>98.0%), hexanoic acid (>99.5%), 2-methylvaleric acid (>98.0%), lithium iodide (LiI, 99.99%), succinic acid (99.5%), 1-iodocyclohexane (stabilized with copper chip, >97.0%), 1,3-dimethyl-2-imidazolidinone (DMI, ≥99.0%) and 1-methyl-2-pyrrolidone (NMP, 99%) were provided by Aladdin. Iridium(IV) iodide (IrI_4_, 99.95% (metals basis), Ir ≥ 27.0%), iridium (III) 2,4-pentanedionate (Ir(acac)_3_, Ir 37.5% min), iridium (III) chloride (IrCl_3_, anhydrous, 99.99% (metals basis)), iridium(IV) oxide dihydrate (IrO_2_·2H_2_O, 99.99% (metals basis)), carbonylchlorobis(triphenylphosphine)iridium (Ir(CO)(PPh_3_)_2_Cl), nickel(II) iodide (NiI_2_, 99.5% (metals basis)), cobalt(II) iodide (CoI_2_, 99.5% (metals basis), anhydrous) and iron(II) iodide (FeI_2_, 97% (metals basis), anhydrous) were obtained from Alfa Aesar China Co., Ltd. (Ward Hill, MA, USA). Trifluoroacetic acid (99%) was provided by J&K Scientific Ltd. (Beijing, China), and lithium chloride (LiCl, 98%) and methyl iodide (CH_3_I, >99.5%) were bought from TCI Shanghai Co., Ltd. (Shanghai, China). Tetrairidium dodecacabonyl (Ir_4_(CO)_12_, 98%) was bought from Sigma-Aldrich Co, LLC. (St. Louis, MO, USA). Palladium(II) iodide (PdI_2_, 99.99%, Pd: 29%) and lithium bromide (LiBr, 99%) were purchased from Beijing InnoChem Science & Technology Co., Ltd. (Beijing, China). Iridium(III) acetate (Ir(OAc)_3_, >97%, Ir ≥ 48.0%) and dicarbonyl(acetylacetonato)iridium(I) (Ir(acac)(CO)_2_, >99%) were purchased from Shanghai Haohong Biomedical Technology Co., Ltd. (Shanghai, China). Sulfuric acid (H_2_SO_4_, 95–98%) and propionic acid (CH_3_CH_2_COOH, ≥99.5%) were obtained from Sinopharm Chemical Reagent Co., Ltd. (Shanghai, China). Acetic acid(≥99.5%) was bought from Concord Technology (Tianjin) Co., Ltd. (Tianjin, China). 2-Ethylpentanoic acid(95%) was purchased from Bide Pharmatech Ltd. (Shanghai, China). CO_2_ (99.99%), H_2_ (99.99%) and CO (99.99%) were provided by Beijing Analytical Instrument Company (Beijing, China). Deuterium gas (D_2_, 99.999%) was offered by Zhengzhou Xingdao Chemical Technology Co., Ltd. (Zhengzhou, China). Carbon dioxide-^13^C (^13^CO_2_, 99% ^13^C) was obtained from Beijing Gaisi Chemical Gases Center (Beijing, China). 

### 3.2. The Catalytic Reaction

The reaction was executed in a stainless steel reactor of 16 mL inner volume and 18 mm inner diameter, which was lined with PTFE and equipped with a magnetic stirrer. In a typical experiment, appropriate amounts of Ir(acac)(CO)_2_ catalyst, LiI promoter, acetic acid solvent and cyclohexene substrate were put one by one into the reactor. The reactor was closed and purged two times with 0.5 MPa CO_2_, and then specific pressures of CO_2_ and H_2_ were sequentially charged into the reactor at room temperature. The temperature of the reactor was enhanced to and maintained at a desired value, stirring at 800 rpm. When the specified reaction time was over, the reactor was quenched in an ice-water bath. The residual gas in the reactor was slowly released and collected in a gas bag for GC analysis. Then, the reactor was opened, and liquid sample was directly taken out to analyze the liquid products and intermediates generated during the reaction. We would like to mention that at the optimized condition, 5.3 MPa CO_2_ and 1 MPa H_2_ were charged to the reactor at room temperature, which inflated to 12.3 MPa at 170 °C. So, these high-pressure experiments should be conducted with extreme caution.

### 3.3. Analysis Methods

The products and intermediates generated during the reactions were identified by GC-MS (Shimadzu GCMS-QP2010, Shimadzu, Kyoto, Japan) with a Rtx-WAX column (30 m in length, 0.32 mm in diameter, 0.25 μm of membrane), which were in contrast to the standards in the LC or GC traces. The gas products were analyzed by a gas chromatograph (Agilent 7890B, Agilent Technologies, Santa Clara, CA, USA) with a packed column (TDX-01, 3 mm in diameter, 1 m in length) and a TCD detector (Shimadzu, Kyoto, Japan), where argon was used as carrier gas. The amount of carboxylic acids in the reaction liquid was tested by a liquid chromatography (LC-10AT, Shimadzu) with a carbohydrate column BP-800H + (Benson polymer, Delhi, India, S/N 23757) and a refractive index detector (RID). The column was kept at 50 °C. The column was eluted with 5 mmol/L H_2_SO_4_ solution at a flow rate of 0.4 mL/min or 0.8 mL/min. Before LC analysis, the reaction solution was diluted with 5 mL of 1/1 AcOH/H_2_O, where succinic acid was utilized as the internal standard. A small amount of this liquid mixture was filtered by a syringe filter with a hydrophilic PTFE membrane (Green Mall, Taizhou, China), and the obtained filtrate was directly injected into the LC. The ^13^C NMR characterization of the reaction liquids was conducted on an NMR spectrometer (Bruker Avance III 400 HD, Bruker, Billerica, MA, USA). High-resolution electrospray ionization mass spectrometry (HR-ESI-MS) was carried out on a Bruker FT-ICR-MS (Solarix 9.4 t). Infrared (IR) spectra were obtained by a Bruker Invenio-S spectrometer (Bruker, Billerica, MA, USA).

## 4. Conclusions

In summary, we developed a new catalyst to produce C_2+_ carboxylic acids via the hydrocarboxylation of olefins with CO_2_ and H_2_. The catalytic system consisting of Ir(acac)(CO)_2_ and LiI could efficiently promote the reaction at 170 °C in an acetic acid solvent. The catalytic system was not only effective for cyclic and linear olefins but also effective for terminal and internal olefins; in addition, it could transform diene to corresponding carboxylic acids. The catalytic system may simultaneously activate the olefin substrates and accelerate the RWGS reaction of CO_2_ and H_2_ to generate CO; subsequently, the organic iodides derived from olefins react with CO via carbonylation to produce the desired carboxylic acids. This paper offers a new protocol for CO_2_ valorization and carboxylic acid fabrication.

## Figures and Tables

**Table 1:** Different catalytic systems for synthesis of ChA from cyclohexene, CO_2_ and H_2_.

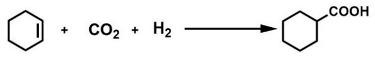				
**Entry**	**Catalyst**	**Promoter**	**Solvent**	**Yield** **(%)** ^**a**^
1	Ir(acac)(CO)_2_	LiI	AcOH	62.8
2	Ir(OAc)_3_	LiI	AcOH	42.3
3	IrI_4_	LiI	AcOH	32.7
4	Ir_4_(CO)_12_	LiI	AcOH	43.0
5	Ir(acac)_3_	LiI	AcOH	44.5
6	IrCl_3_	LiI	AcOH	37.6
7	IrO_2_·2H_2_O	LiI	AcOH	0
8	IrCl(CO)(PPh_3_)_2_	LiI	AcOH	0
9	Ir(acac)(CO)_2_	I_2_	AcOH	0
10	Ir(acac)(CO)_2_	LiCl	AcOH	0
11	Ir(acac)(CO)_2_	LiBr	AcOH	0
12	Ir(acac)(CO)_2_	NaI	AcOH	10.3
13	Ir(acac)(CO)_2_	KI	AcOH	0
14	Ir(acac)(CO)_2_	CH_3_I	AcOH	7.2
15	Ir(acac)(CO)_2_	LiI	CH_3_CH_2_COOH	45.3

Reaction conditions: 60 μmol catalyst (based on metal), 1.25 mmol promoter, 0.6 mL solvent, 1 mmol cyclohexene, 5.3 MPa (68 mmol) CO_2_, 1.0 MPa H_2_ (at room temperature), 170 °C, 14 h. ^a^ The yield was based on cyclohexene, which was calculated based on the data from liquid chromatography.

**Table 2:** The catalytic results with CO instead of CO_2_ and H_2_.

Entry	CO [MPa]	H_2_ [MPa]	Yield (%)
1	0.5	0	72.3
2	1	0	59.4
3	2	0	36.5

Note: Other conditions were the same as those in entry 1 of Table 1.

**Table 3:** The reaction results using the organic intermediates and different gases.

Entry	Intermediate	Product	Yield (%)
1	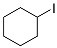	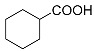	36.6 ^a^
			34.5 ^b^
2	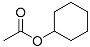		45.0 ^a^
			42.7 ^b^

The gases charged at room temperature: ^a^ 5.3 MPa (68 mmol) CO_2_ and 1.0 MPa H_2_, ^b^ 0.5 MPa CO. Other conditions were the same as those in entry 1 of Table 1.

**Table 4:** Synthesis of carboxylic acids via reaction of CO_2_ and H_2_ with different olefins ^[a]^.

Entry	Substrate	Product	Yield (%) ^[b]^
1	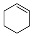	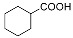	62.8
2	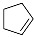	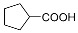	32.0
3	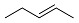	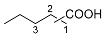	49.8 ^[c]^
4	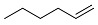	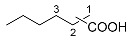	45.1 ^[d]^
5	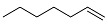	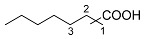	40.7 ^[e]^
6	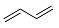	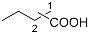 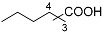	36.7 ^[f]^
7	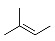	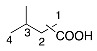	17 ^[g]^

Notes: ^[a]^ Other conditions were the same as those of entry 1 in Table 1. ^[b]^ The yield was based on the olefin substrate, which was determined by liquid chromatography. ^[c]^ 1:2:3 = 46:29:25. ^[d]^ 1:2:3 = 49:26:25. ^[e]^ 1:2:3 = 55:26:19. ^[f]^ 1:2:3:4 = 54:24:13:9. ^[g]^ 1:2:3:4 = 31:10:2:57.

## Data Availability

The original contributions presented in this study are included in the article/Supplementary Materials. Further inquiries can be directed to the corresponding authors.

## Supplementary Materials

The following supporting information can be downloaded at https://www.mdpi.com/article/10.3390/molecules30071599/s1, Figure S1: The LC graph of the liquid sample after the reaction in entry 1 of Table 1; Figure S2: The GC-MS graph of the liquid sample after the reaction in entry 1 of Table 1; Figure S3: The GC graph of the gaseous sample after the reaction in entry 1 of Table 1; Figure S4: The GC-MS graph of the liquid sample after 1 h of the reaction in entry 1 of Table 1; Figure S5: The GC graph of the gaseous sample at 1 h of the reaction in entry 1 of Table 1; Figure S6: The GC-MS graph of the liquid sample after the ^13^CO_2_ labeling test; Figure S7: The GC-MS graph of the liquid sample after the H_2_^18^O labeling test; Figure S8: The GC-MS graph of the liquid sample after the D_2_ labeling test; Table S1: The price of the representative substrates and products; Table S2: Effect of the catalyst precursors on the reaction of cyclohexene, CO_2_ and H_2_; Table S3: Effect of the solvents on the reaction of cyclohexene, CO_2_ and H_2_; Table S4: Influence of catalyst dosage on the reaction of cyclohexene, CO_2_ and H_2_; Table S5: Influence of promoter dosage on the reaction of cyclohexene, CO_2_ and H_2_; Table S6: Influence of solvent volume on the reaction of cyclohexene, CO_2_ and H_2_; Table S7: Effect of gases pressures on the reaction of cyclohexene, CO_2_ and H_2_; Table S8: The yield of the reaction of cyclohexene, CO_2_ and H_2_ at different time.
