# Supplementary material for: Iridium-Catalyzed Hydrocarboxylation of Olefins with CO2 and H2
Source: Molecules. 2025 Apr 3;30(7):1599. doi: 10.3390/molecules30071599 (PMC11990289; doi:10.3390/molecules30071599)
Supplement: Supplementary file 1 [file molecules-30-01599-s001.zip › molecules-3508288-supplementary.pdf]

Supplementary Information

Iridium-Catalyzed Hydrocarboxylation of Olefins with CO_2 and H_2

Yang Li, et al.

Figures and Tables

1. Figures

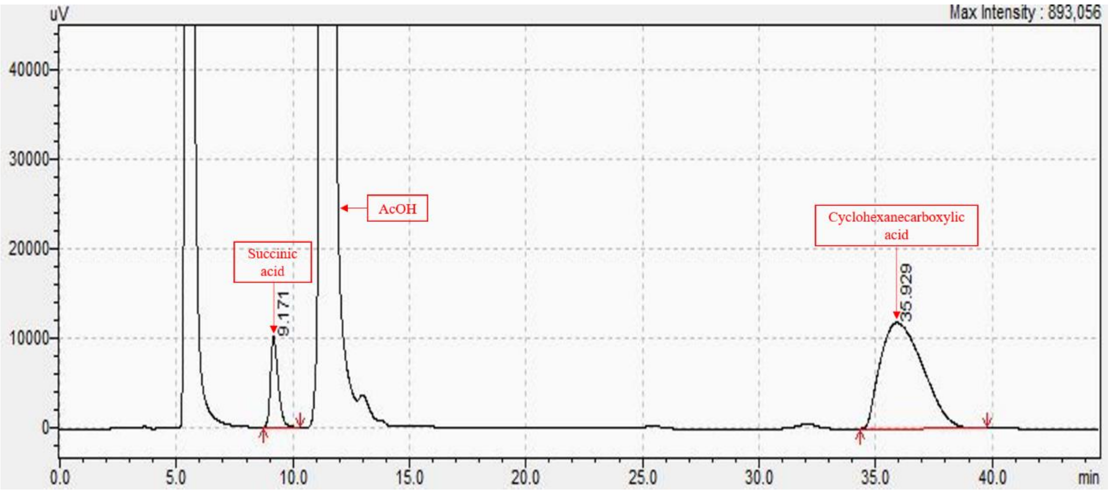

Figure S1. The LC graph of the liquid sample after the reaction in entry 1 of Table 1. For LC analysis, the liquid sample was diluted with AcOH and H_2O, where succinic acid was used as the internal standard.

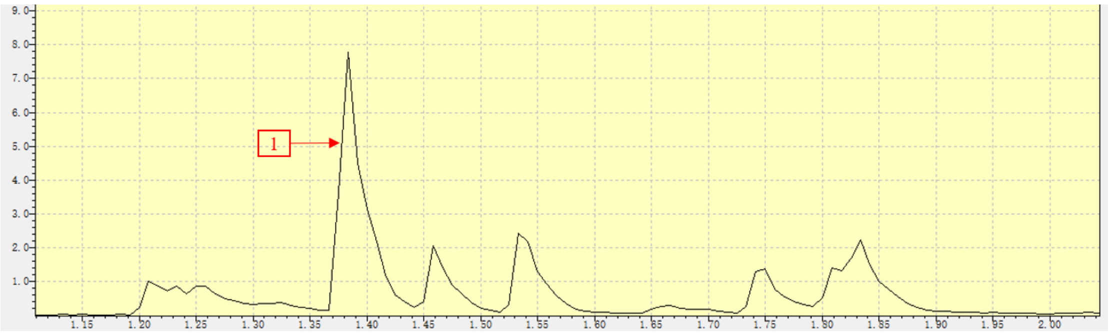

Target 1

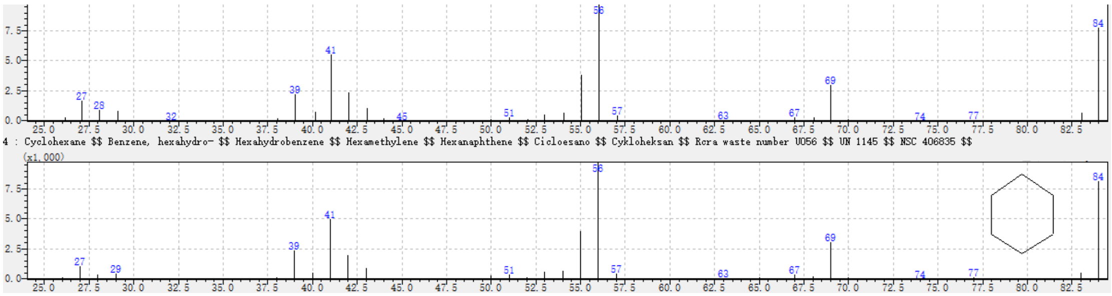

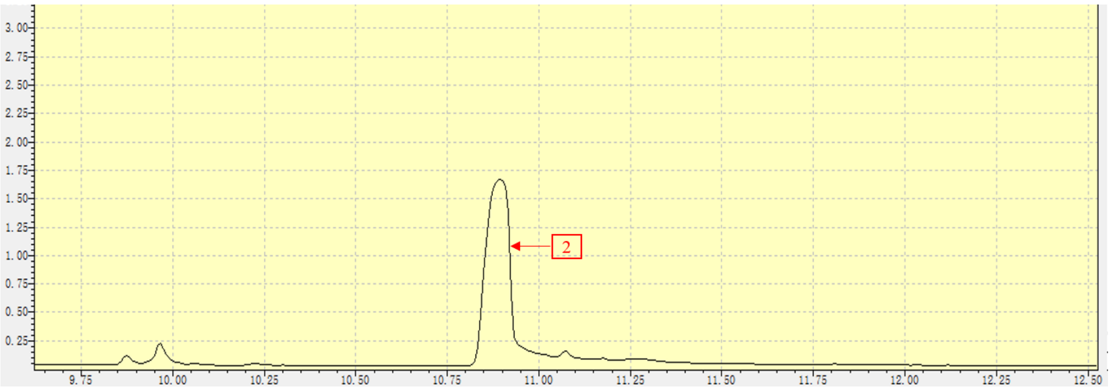

Target 2

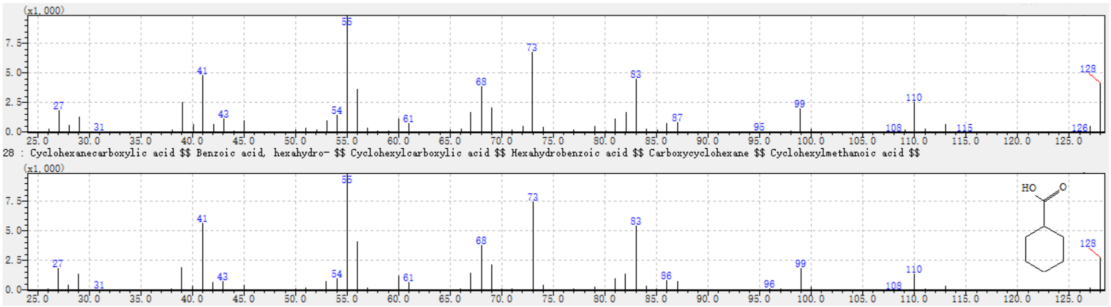

Figure S2. The GC-MS graph of the liquid sample after the reaction in entry 1 of Table 1.

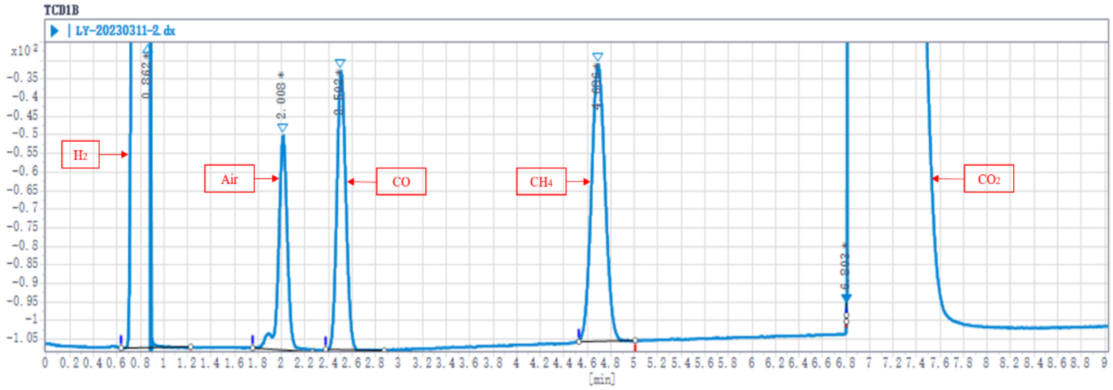

Figure S3. The GC graph of the gaseous sample after the reaction in entry 1 of Table 1.

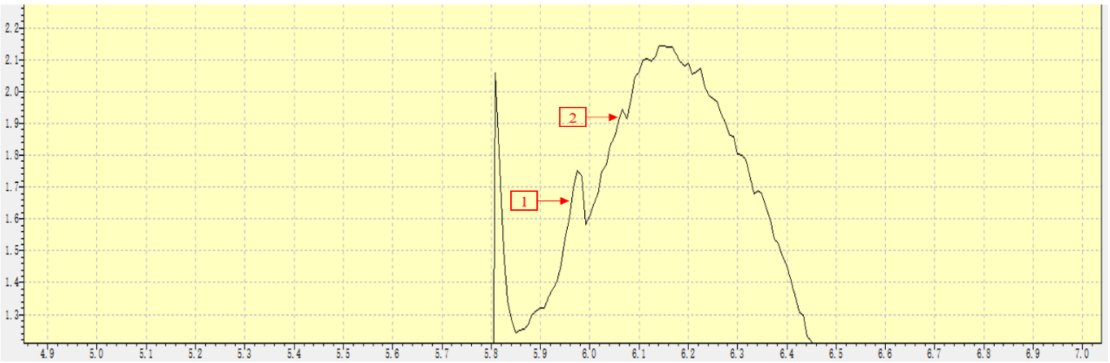

Target 1

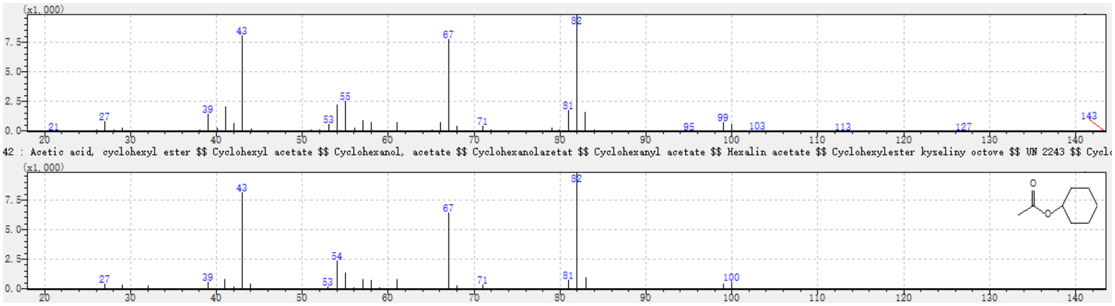

Target 2

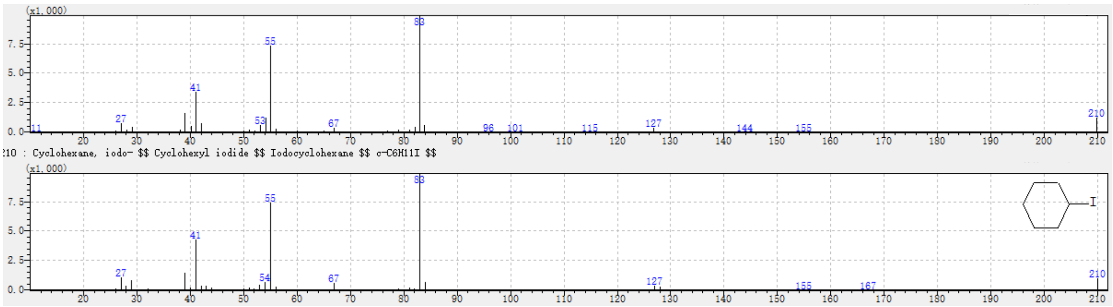

Figure S4. The GC-MS graph of the liquid sample after **1 h** of the reaction in entry 1 of Table 1.

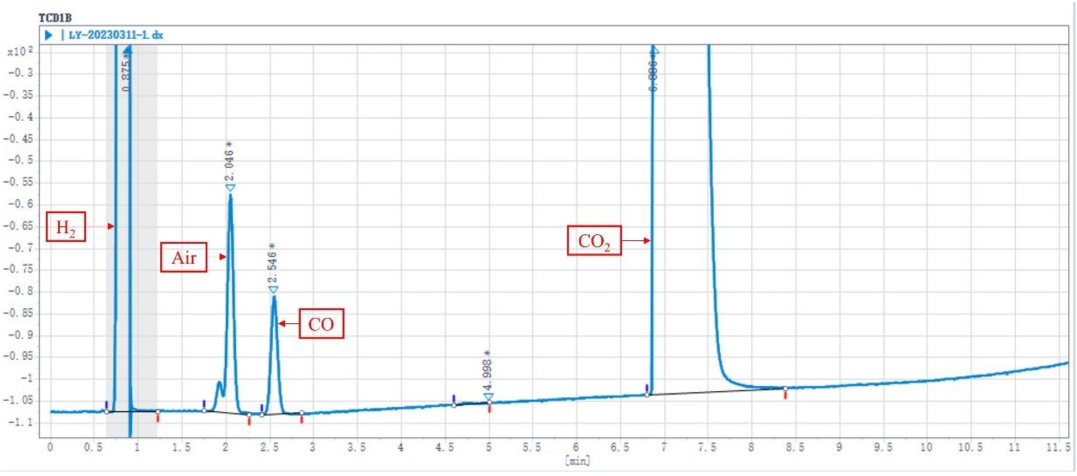

Figure S5. The GC graph of the gaseous sample at **1 h** of the reaction in entry 1 of Table 1.

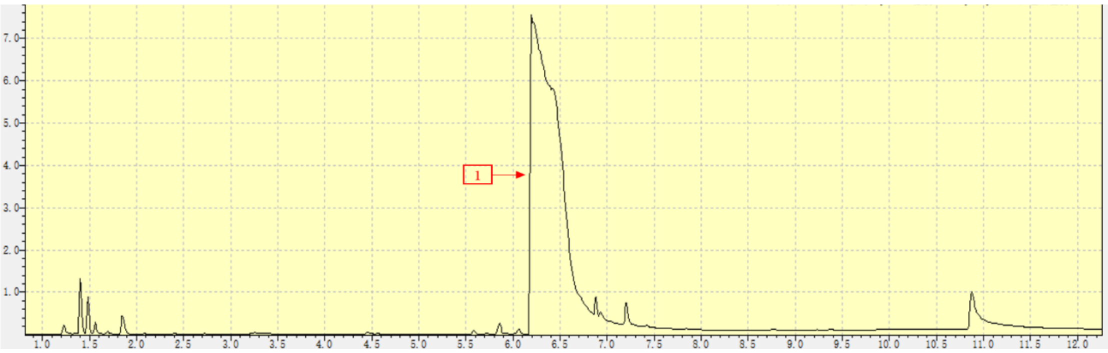

Target 1

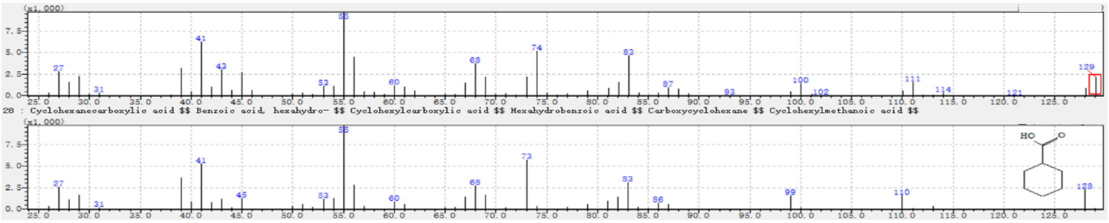

Figure S6. The GC-MS graph of the liquid sample after the $^{13}CO_2$ labelling test. The conditions were the same as those of entry 1 in Table 1 except that 4 MPa $^{13}CO_2$ was used instead of CO_2.

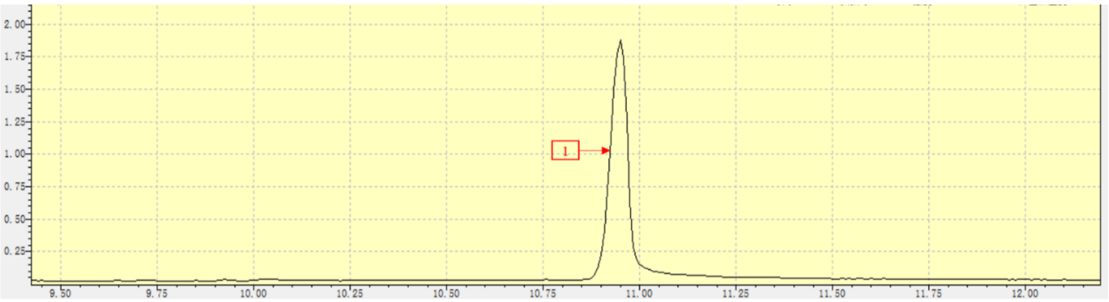

Target 1

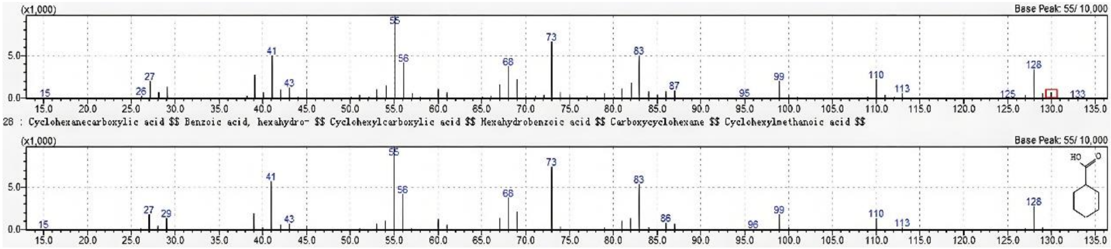

Figure S7. The GC-MS graph of the liquid sample after the $H_2{}^{18}O$ labelling test. The conditions were the same as those of entry 1 in Table 1 except that 0.25 mL $H_2{}^{18}O$ was added.

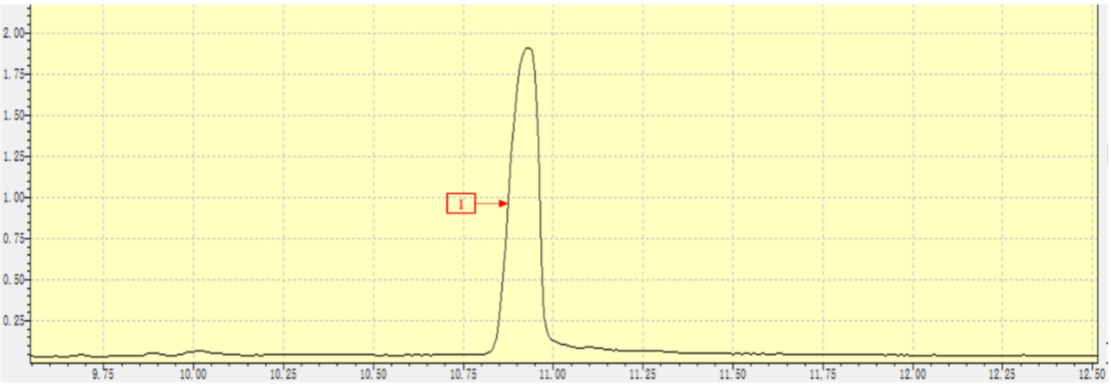

Target 1

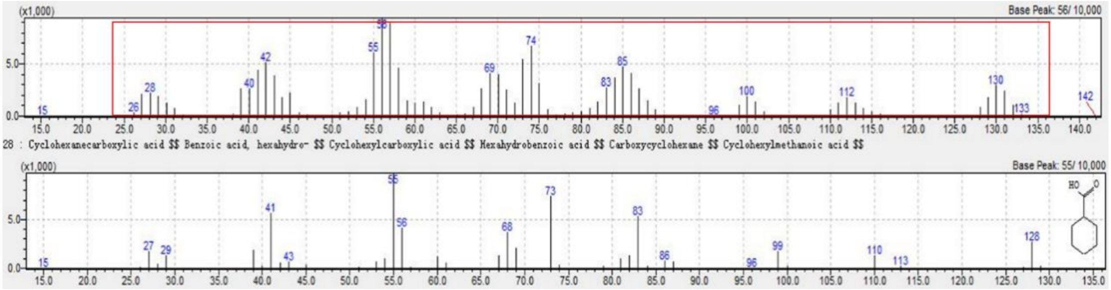

Figure S8. The GC-MS graph of the liquid sample after the D_2 labelling test. The conditions were the same as those of entry 1 in Table 1 except that D_2 was used instead of H_2.

2. Tables

Table S1. The price of the representative substrates and products.

Substrates	Products
Cyclohexene,¥77.59/L (Aladdin)	ChA, ¥251.04/Kg (Aladdin)

The above price information was obtained from https://www.inno-chem.com.cn/ in May, 2024.

Table S2. Effect of the catalyst precursors on the reaction of cyclohexene, CO_2 and H_2.

Entry	Catalyst	Promoter	Solvent	Yield (%)
1	FeI_2	LiI	AcOH	0
2	CoI_2	LiI	AcOH	0
3	NiI_2	LiI	AcOH	0
4	RhI_3	LiI	AcOH	0
5	PdI_2	LiI	AcOH	0

Note: 60 μmol catalyst (based on metal) was used in the reaction, and other conditions were the same as those in entry 1 of Table 1.

Table S3. Effect of the solvents on the reaction of cyclohexene, CO_2 and H_2.

Entry	Catalyst	Promoter	Solvent	Yield (%)
1	$Ir(acac)(CO)_2$	LiI	H_2O	0
2	$Ir(acac)(CO)_2$	LiI	DMSO	0
3	$Ir(acac)(CO)_2$	LiI	DMI	0
4	$Ir(acac)(CO)_2$	LiI	NMP	0
5	$Ir(acac)(CO)_2$	LiI	HCl(aq)	0
6	$Ir(acac)(CO)_2$	LiI	H_2SO_4(aq)	0
7	$Ir(acac)(CO)_2$	LiI	HCl(aq)/NMP	2.6
8	$Ir(acac)(CO)_2$	LiI	CF_3COOH/H_2O	2.1

Note: 0.6 mL solvent was used in the reaction, and other conditions were the same as those in entry 1 of Table 1. The acidities of acetic acid and the mixed solvents were tested by the pH indicator paper, and the results are shown below.

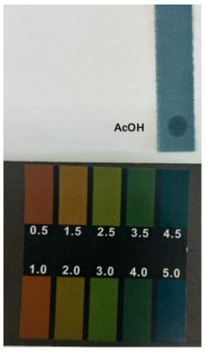

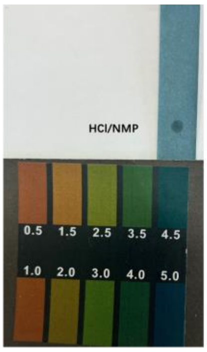

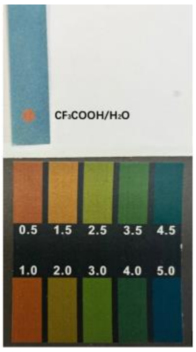

Acetic acid HCl(aq)/NMP CF_3COOH/H_2O

Table S4. Influence of catalyst dosage on the reaction of cyclohexene, CO_2 and H_2.

Catalyst	Promoter	Solvent	Catalyst amount (μmol)	Yield (%)
$Ir(acac)(CO)_2$	LiI	AcOH	45	41.8
$Ir(acac)(CO)_2$	LiI	AcOH	60	62.8
$Ir(acac)(CO)_2$	LiI	AcOH	75	48.7

Note: Other conditions were the same as those in entry 1 of Table 1.

Table S5. Influence of promoter dosage on the reaction of cyclohexene, CO_2 and H_2.

Catalyst	Promoter	Solvent	Promoter amount (mmol)	Yield (%)
$Ir(acac)(CO)_2$	LiI	AcOH	1	54.7
$Ir(acac)(CO)_2$	LiI	AcOH	1.25	62.8
$Ir(acac)(CO)_2$	LiI	AcOH	1.5	48.4
$Ir(acac)(CO)_2$	LiI	AcOH	2	46.3
$Ir(acac)(CO)_2$	LiI	AcOH	2.25	40.5

Note: Other conditions were the same as those in entry 1 of Table 1.

Table S6. Influence of solvent volume on the reaction of cyclohexene, CO_2 and H_2.

Catalyst	Promoter	Solvent	Solvent volume (mL)	Yield (%)
$Ir(acac)(CO)_2$	LiI	AcOH	0.4	50.7
$Ir(acac)(CO)_2$	LiI	AcOH	0.6	62.8
$Ir(acac)(CO)_2$	LiI	AcOH	0.8	50.1

Note: Other conditions were the same as those in entry 1 of Table 1.

Table S7. Effect of gases pressures on the reaction of cyclohexene, CO_2 and H_2.

CO_2 (MPa)	H_2 (MPa)	Yield (%)
5.3	3	28.0
5.3	1	62.8
4	1	22.8
4	0	0
0	4	0

Note: The gases were charged at room temperature. Other conditions were the same as those in entry 1 of Table 1.

Table S8. The yield of the reaction of cyclohexene, CO_2 and H_2 at different time.

Catalyst	Promoter	Solvent	Time (h)	Yield (%)
$Ir(acac)(CO)_2$	I_2	AcOH	10	41.1
$Ir(acac)(CO)_2$	I_2	AcOH	12	48.0
$Ir(acac)(CO)_2$	I_2	AcOH	14	62.8
$Ir(acac)(CO)_2$	I_2	AcOH	16	63.3

Note: Other conditions were the same as those in entry 1 of Table 1.
